# Clinical and Functional Outcome of Meniscal Injuries Treated with Platelet-Rich Plasma: A Single-Center Case Series

**DOI:** 10.3390/ijerph19127118

**Published:** 2022-06-10

**Authors:** Ivan Medina-Porqueres, Pablo Martin-Garcia, Sofia Sanz-De-Diego, Abel Gomez-Caceres, Francisco Moya-Torrecilla, Marcelo Reyes-Eldblom, Daniel Rosado-Velazquez

**Affiliations:** 1 Department of Physical Therapy, Faculty of Health Sciences, University of Malaga, 29071 Malaga, Spain; 2 Malaga Football Club, Medical Services, 29011 Malaga, Spain; gomezcaceresabel@gmail.com (A.G.-C.); dr_rosado2002@hotmail.com (D.R.-V.); 3 Onco-Hematology Unit, University Hospital Virgen de la Victoria, 29010 Malaga, Spain; martingarciapablo@hotmail.com; 4 Puerperium Unit, Regional Hospital of Malaga, 29011 Malaga, Spain; sofiasanzdediego@gmail.com; 5 Vithas Xanit International Hospital, 29630 Benalmadena, Spain; franmoya57@hotmail.com; 6 Costa del Sol Medical Center, 29620 Torremolinos, Spain; mreldblom@gmail.com

**Keywords:** meniscus injury, growth factors, perimeniscal, knee

## Abstract

Meniscal injuries are among the most frequently encountered conditions in the knee joint. Therapeutic approaches are diverse and are largely dependent on the extent and location of the injury. The purpose of this study was to describe the clinical and functional outcomes of an intraarticular and percutaneous platelet-rich plasma (PRP) injection regime in patients with stable meniscal injuries. Demographics, the type of tear, affected knee, surgical procedure, type of intervention, follow-up period, and outcomes were recorded in all cases. Patient-reported outcome measures included the Knee Injury and Osteoarthritis Outcome Score (KOOS) and Tegner activity level scale. Overall patient satisfaction, quality of life, and pain intensity were also assessed. A total of 38 cases (8 females) had sustained a stable meniscal lesion (32 medial, 6 lateral) and met the inclusion criteria. All of them received three intraarticular and percutaneous PRP injections. Patients receiving the PRP injection regime reported clinically (*p* = 0.000) and functionally (*p* = 0.000 and *p* = 0.001) significant improvement in all outcome measures during this interval. All patients reported they were very satisfied or satisfied with the outcome. The results of this study suggest that the treatment of stable meniscal injuries with percutaneous–intraarticular PRP injections can achieve a significant clinical and functional improvement.

## 1. Introduction

Meniscal injuries are among the most frequently encountered and treated conditions in the knee joint and are responsible for clinical and functional impairment, with a relatively high annual cost [1]. Meniscus lesions are also common in young athletes, particularly in contact sports, with a mean annual incidence of approximately 60–70 per 100,000 [2]. When the integrity of the meniscus is altered, contact stresses within the knee joint are increased. Moreover, previous research has established a strong relationship between meniscal tear and cartilage deterioration [3,4].

Therefore, adequate treatment strategies are required to prevent these potential complications. Various therapeutic approaches have been described; these are largely dependent on the extent and location of the injury. The menisci have a unique vascular anatomy: only their peripheral third sustains a complete vascular supply [5]. Thus, the therapeutic results vary significantly depending on the specific tear pattern. Arthroscopic meniscal repair and meniscectomy have traditionally been the most commonly used treatment methods [3]. However, different health authorities have recommended against the use of knee arthroscopy in patients aged ≥40 years with knee osteoarthritis (KOA) [6]. 

In recent years there has been growing interest in the preservation of meniscal integrity in both isolated and concomitant knee injuries. Accordingly, a more conservative approach for the treatment of meniscal injuries has been recommended in recent decades, especially in stable or degenerative meniscal injuries, as arthroscopic repair or meniscectomy may also increase the risk of KOA [4].

Platelet-rich plasma (PRP) is an autologous blood product obtained through the centrifugation of whole blood, therefore increasing the concentration of platelets that is believed to be 3 to 6 times higher than baseline values [7]. The physiological role of platelets in the natural healing process has led to the development of several research fields focusing on them as the main therapy for a variety of musculoskeletal conditions, particularly in certain cartilage pathologies [8,9]. There has been a spreading interest in the application of PRP in the treatment of meniscal injuries in recent years; however, only a few studies have been reported [10,11,12,13,14]. Fibrochondrocyte migration and extracellular matrix production have been demonstrated to be positively affected by PRP cytokines in vitro [15,16]. Clinically, the use of PRP within the meniscal tear [11,17] and the surrounding tissues [18] has shown positive results. On the other hand, the results on the application of PRP during arthroscopic meniscal repairs have been inconclusive [13,14,19,20]. Nevertheless, the effect of a combined intraarticular and percutaneous PRP injection in stable meniscal injuries has not been studied previously. Accordingly, the purpose of this study was to determine the clinical and functional outcomes of patients with stable meniscal injuries treated with intraarticular and percutaneous PRP injections.

## 2. Materials and Methods

This study was retrospectively registered at ClinicalTrials.gov (NCT04040283). The study was performed in accordance with the Declaration of Helsinki as well as the International Conference on Harmonization: Guidelines for Good Clinical Practice. The study was approved by our Institutional Review Board (n. 1-2020-H). All patients gave their consent to allow their clinical and radiological data to be used for research purposes. 

### 2.1. Patient Selection

Data were withdrawn from our electronic medical record system. We retrospectively identified all patients attending our institution with a diagnosis of meniscal lesion confirmed by magnetic resonance imaging (MRI) and who were treated with a PRP injection between May 2015 and December 2019. Patients with meniscus injuries who had failed conservative treatment after a minimum period of 6 weeks were included. The following conservative therapies before PRP therapy were accepted: stretching, orthosis, physiotherapy, therapeutic exercise, oral analgesia, electrothermal therapy, and extracorporeal shock wave therapy. These conservative therapies were not monitored by our researchers since all patients were subjected to this conservative approach before consulting our institution.

The following information was collected: demographic data, past medical history, diagnostic imaging, meniscus injury pattern, previous surgical interventions, and details about subsequent revisions. Meniscus injuries were classified according to Reicher et al., who defined four grades on MRI: Grade I (*no tear*) are defined as homogenously black meniscus; Grade II (*unlikely tear*), with a region of minimally increased signal intensity within the meniscus, usually not present on two adjacent scans; Grade III (*probable tear*), with a small, linear region of increased signal intensity/a small-to-moderate nonlinear area of increased signal intensity within the meniscus; Grade IV (*definite tear*), with gross distortion of the normal shape, truncation of the meniscus/a large focus or line of increased signal intensity within the meniscus [1]. For the purpose of this retrospective analysis, Grades I–III were considered as stable injuries since neither gross distortion nor truncation of the meniscal material existed in these conditions. Patients subjected to previous steroid/hyaluronic intraarticular injections, a history of complex/unstable meniscal tears [21]—displaced meniscal fragment, presence of fluid signal intensity within the tear on T2-weighted images, tear > 10 mm in length, and tears with complex patterns—, treated with nonsteroidal antiinflammatory drugs (NSAIDs) within 2 days of the PRP injection, with local infections at the site of the procedure, or who underwent knee surgery were excluded. In addition, individuals suffering from severe diabetes or hyperuricemia, bleeding disorders, severe cardiovascular disease, or those who were receiving anticoagulant treatment were also discarded. Pregnant women and individuals treated with systemic corticosteroid or subjected to a previous corticosteroid injection at the affected site within 1 month of the PRP injection were also excluded.

### 2.2. Platelet-Rich Plasma Injection

A total of 45 mL whole blood was collected from the medial cubital vein of every patient. Then, the blood underwent a standardized protocol of preparation, which consisted of one centrifugation (Auxilab, Nahita Blue, FugelabGB10, Navarra, Spain) at 1800 rpm for 8 min. Each patient received the same 6 mL intraarticular injection into the affected knee. The injection site on the skin was prepped under aseptic conditions with povidone-iodine solution. Patients were placed in a supine position with knee flexion of 90 degrees, and a lateral approach to the knee was used. PRP was injected intraarticularly using a 22-gauge needle with classic infrapatellar approach [22,23]. Percutaneous PRP application was performed based on previous described approaches [18,24]. A 25-gauge needle was advanced until the meniscus wall was reached. Once the needle touched this wall it was retracted by 1 mm and an injection of 0.5 mL of PRP around perimeniscal tissues was performed, using the abovementioned strict aseptic administration technique. This procedure was repeated 2–3 times into the adjacent perimeniscal aspect, with a total 1.5–2 mL of PRP injected. Then, the patients were asked to mobilize the knee to facilitate the dissemination of the PRP. A total of 3 injections were administered on seven-day intervals during a three-week period. The injection point was determined by the identification of anatomical landmarks. The tibial plateau and femoral notch were used as references to localize the joint line. Meniscal tissue was identified by increase and loss of resistance across the joint line, which corresponds to meniscus “red zone” [25]. No local anesthetic was administered before injection to prevent a possible negative interaction due to its potential negative influence on PRP effectiveness through pH modifications [26]. All the practitioners who performed PRP injection treatment in our institution followed the same protocol regarding preparation and maintenance of aseptic technique. Patients were instructed to avoid the use of NSAIDs during treatment up to 2 weeks after the last injection, to prevent possible negative interactions with the PRP. Paracetamol was recommended as an analgesic treatment when needed. Participants were advised to apply ice routinely and were allowed to fully weight bear and to resume their daily living activities. However, patients were advised to avoid impact activities such as running and jumping up to 2 weeks after the last PRP injection. A gradual increase in weightbearing/impact activities was recommended over the following 4–8 weeks. Return to sport was allowed on the individual’s tolerance and requirements.

### 2.3. Clinical and Functional Evaluation

The clinical and functional evaluation were considered the primary outcomes of this study. Clinical improvement was measured by Numeric Pain Rating Scale (NPRS) and the Feeling Thermometer Scale. Functional capacities were assessed using the Knee Injury and Osteoarthritis Outcome Score (KOOS) and Tegner activity scale. Patients’ satisfaction with PRP therapy was used as a secondary outcome. Furthermore, patients were asked if they would receive the PRP procedure again in the same clinical situation. The NPRS indicates the subjective feeling of pain, with 0 indicating no pain and 100 indicating the worst pain the patient has ever experience [27]. The KOOS is a reliable, validated, and reproducible patient-reported outcome employed to evaluate the symptoms and function of patients with knee ailments [28,29]. This 42-item self-report questionnaire is comprised by five different subscales, “Symptoms”, “Pain”, “Activities of Daily Living” (ADL), “Sport”, and “Quality Of Life” (QoL). The result is a percentage score that ranges from 0 to 100 (0 = extreme knee problems, 100 = no knee problem) [30]. Differences of 8 points and over have been considered to be clinically relevant [31]. The Feeling Thermometer is a visual analog scale from 0 to 100 that has previously been employed as a valid measure of health status in clinical research due to its ease of administration and simplicity [32]. The Tegner activity scale is a one-item score based on daily activities, recreation, and competitive sports. The patients select the level of participation that best describes their current level of activity. A score of 0 implies “sick leave or disability pension because of knee problems”, whereas a score of 10 corresponds to participation in national and international elite competitive sports. Those participating in recreational or competitive sport will select activity levels between 6 and 10 [33]. Patients’ overall satisfaction with PRP therapy was evaluated by asking the patients if they were very satisfied, satisfied, partially satisfied, or unsatisfied. Additionally, a 10-point NPRS for pain was used [34].

### 2.4. Statistical Analysis

Statistical analysis was performed using SPSS statistics, version 25 (IBM SPSS, Armonk, NY, USA) to compare clinical and functional outcomes between pre-intervention and post-intervention groups. Continuous variables were presented as means with their corresponding standard deviations (SD). The distribution of continuous variables was analyzed using the Shapiro–Wilk test. Treatment safety and efficacy were assessed by comparing the pre- and post-treatment scores using paired-samples *t*-test. A *p*-value < 0.05 was considered statistically significant for all comparisons.

## 3. Results

During the study period, we identified a total of 38 patients with stable meniscal injuries (i.e., 32 medial and 6 lateral meniscal injuries) who met our inclusion criteria. The mean patient age at PRP intervention was 50.68 ± 9.65 years (range, 29–72 years) and the mean body mass index (BMI) was 25.77 ± 3.37 kg/m^2^ (range, 17.63–33.64 kg/m^2^). The mean follow-up period after PRP therapy was 75.92 ± 31.7 days (range, 39–190 days). The left knee was affected in 13 patients (31.6%). We observed that the most common meniscal tear type was Grade III injury (*n* = 31; 81.57%). No bilateral injuries were identified in our series. The demographic features of the study group are presented in Table 1. More than half of the 38 evaluated patients (*n* = 31; 81.57%) regularly practiced recreational sports (i.e., eight gym, five soccer, four tennis/paddle, three cross-fit, and three yoga) at an amateur level. In terms of physical workload, no heavy physical workload (i.e., manual laborers or construction workers) was reported. However, a small group of patients (*n* = 3, 7.89%) had occupations requiring an average workload (i.e., electricians or plumbers).

Table 2 gathers in detail the clinical and functional outcomes of each patient along with the subjective overall satisfaction. The average baseline and post-treatment NPRS scores were 5.86 ± 1.91 and 1.59 ± 1.36 (*p* = 0.00), respectively, whereas the mean baseline and post-treatment Feeling Thermometer values were 67.89 ± 16.42 and 86.31 ± 6.74 (*p* = 0.00), respectively.

The participants experienced a significant improvement in the total KOOS score (i.e., 41.89 ± 22.89 baseline and 85.94 ± 13.5 post-treatment, (*p* = 0.000)). The post-treatment KOOS subscores also showed good to excellent results: KOOS symptoms 86.36 ± 10.19, KOOS pain 89 ± 12.74, KOOS ADL 90.97 ± 11.89, KOOS 5 Sport/Rec 78.97 ± 22.16, and KOOS QOL 78.72 ± 12.1. The mean Tegner scale also showed as significantly improved (i.e., 3.73 ± 1.67 baseline and 4.73 ± 1.70 post-treatment, (*p* = 0.001)), indicating a moderate level of sports participation (Table 3). All patients were either very satisfied or satisfied with the outcome. None of the patients stated that they would not undergo the same procedure again, or needed additional surgical interventions. No treatment-related adverse effects were reported.

## 4. Discussion

This investigation presents the results referring to the largest series of PRP-treated meniscal injuries sustained by a non-professional athletic population. In the present retrospective study, a positive effect for three PRP intraarticular and percutaneous injections (once a week) was found, with pain reduction, health status amelioration, and function improvement at a mean follow-up of 75.92 days. Our findings seem to confirm the clinical/functional benefit of PRP percutaneous injections for meniscal lesions, as previously described by Blanke et al. for intrasubstance meniscus injury [18].

The most important finding in this present study was that the subjective scores of the KOOS, Tegner, Feeling Thermometer, and NPRS after PRP therapy achieved significant improvements with a patients’ satisfaction rate of 100% (very satisfied/satisfied) at a mean follow-up of 75.92 days. No complications or adverse effects were recorded in our series, highlighting PRP as a reliable and safe alternative to treat stable meniscal lesions and/or to postpone invasive surgical procedures. Thus, these data may add preliminary promising results to the existing knowledge. Accordingly, combined percutaneous and intraarticular PRP injections have a positive influence on clinical and functional outcomes in the context of a meniscal injury. The rationale for this could be explained by the action of different growth factors. Fibroblast growth, platelet-derived, and transforming growth factor-beta (TGF-ß1) factors may play a role in the scar formation process, especially at the red-red zone [10,35]. Moreover, in vitro studies have reported that meniscal cells in the white/white zone may favorably respond to exposure to PDGF-AB [36]. A similar isolated perimeniscal approach has been proposed in the past for treating a cohort of patients with medial meniscus protrusion associated with medial collateral ligament displacement in people with KOA. In this series corticosteroid ultrasound-guided injections were applied with promising clinical results [24].

Meniscal injuries are common and disabling knee conditions, which are commonly advocated for surgical procedures. They also represent an important economic burden due to their high prevalence in the general population [37,38]. Research on conservative approaches for the treatment of meniscal injuries is scarce. Nonoperative choices include rest, immobilization, weightbearing limitation, physical therapy, therapeutic exercise, and intraarticular injections, steroid or hyaluronic acid. No drugs or therapies have proven to result in clinically relevant benefits at 12-months of follow-up in comparison with arthroscopic partial meniscectomy [39]. Recent research has proven the musculoskeletal benefits of PRP on various conditions in sports medicine and orthopedics fields [40,41,42]. Most of the published evidence on PRP has focused on its influence to treat tendon, muscle, and cartilage disorders. However, few attempts have been made regarding the use of PRP for the treatment of meniscal injuries, most of them being isolated case reports [17,18] and short series [11,43,44,45]. Some other attempts have investigated the additive effect of PRP over a previous surgical procedure [13,14,20]. Additionally, animal studies have demonstrated a beneficial effect on the controverted white/white zone when treated with PRP [46]. The additive effect of PRP has been evaluated on open meniscal repair for grade 2–3 horizontal meniscal injuries through a case–control study. PRP improved clinical outcomes when injected into the lesion at the end of the procedure after a standard open meniscal repair, and when compared with this surgical procedure alone [14]. On the other hand, a preclinical study that analyzed the effects of PRP in meniscal tissue regeneration employing a gelatin hydrogel described remarkable healing properties. This investigation focused on the inner avascular part of meniscal injury in vitro and in vivo [10]. However, the independent effect of PRP over injured menisci was not assessed.

A novel aspect in our series was the combination of both intraarticular and percutaneous PRP injections. This combined approach may have multiplied the described potential influence of the PRP product on the fibrocartilage tissue. Others have suggested that this early success of PRP injections may have been related to the reduction in synovial inflammation and the creation of a more balanced intraarticular balance rather than the healing of the meniscus itself [47]. In any case, our findings represent a potential area of research as no previous reports have been published in the scientific literature. Accordingly, this approach could enhance meniscal healing through a more localized action of the PRP. Patients were advised to perform stretching exercises after PRP injections. In addition, a strengthening regime with progressive weightbearing activities was recommended. This regime was based on patients’ tolerance, which was defined as no swelling or pain immediately following or during activities, as well as the day after. 

The standardization of PRP parameters is still not possible with the current techniques and knowledge due to a subject’s own variability in terms of circulating blood products. Different methodologies have been proposed regarding PRP preparation as PRP research have been debating this extreme since its introduction [48,49]. The number of employed platelets or presence of white and red cells may all represent significant outcome parameters. However, our study did not calculate platelet or white/red cell concentrations, which may constitute a significant limitation, so dose–response correlation could not then be established. In any event, analysis of the composition of the injected PRP is only reported in one out of three studies [46]. In parallel, a single centrifugation involving a 2–3 fold increase in platelet concentration from baseline values has been described from PRP therapy origins [50]. Two-stage techniques have also been proposed with acceptable platelet concentrations [42]. Nevertheless, a second spin may decrease platelet viability due to a lytic effect [51]. As with previously published PRP clinical studies, our series involved a single-spin centrifugation protocol.

Surgical management often includes structural modification of the meniscal tissue with obvious implications for the biomechanics of the knee. The resection of these structures increases contact stress on cartilage and accelerates the development of knee compartment osteoarthritis [52]. In addition, meniscectomies might lead to post-surgical complications, as with any given procedure. Potential infections, systemic adverse reactions, cartilage damage, or excessive meniscal resection may occur. Moreover, intra-meniscal lesions cannot be detected arthroscopically in all cases since these injuries do not extend to the articular surface. In this study, clinical and functional improvements were achieved with no significant complications using minimally invasive procedure. For these reasons, we consider that surgical interventions for stable meniscal lesions should be reserved for cases where PRP injections are not effective.

There are some limitations in this study that must be addressed [53]. Firstly, due to the retrospective, observational nature of this study, a causal relationship between the intervention and outcomes could not be established. Secondly, our eligibility criteria were not randomized as we formulated a representative group of patients from our clinical practice, this inherently implies a possible selection bias. Finally, the study population was relatively small, which limits the overall validity of our results. In addition, no imaging follow-up was included in our cohort, and comparisons between the meniscal structure before and after treatment, as in previous reports [11,18,20], were not performed. However, evidence in the literature suggests that asymptomatic knees may hold structurally damaged menisci; however, this clinical scenario is more common in a degenerative setting [54,55]. In our series, the role of PRP in promoting meniscal defect healing was not confirmed as no radiological follow-up was achieved. One may also highlight the absence of imaging guidance for our PRP injections. Previous research has shown no advantages of ultrasound guided infiltrations over direct palpation of the most tender or affected area as guidance for the injection of joint and soft tissue injuries [56,57,58,59].

## 5. Conclusions

This retrospective, single-center case series showed that PRP is a safe and effective treatment modality for stable meniscal injuries through percutaneous and intraarticular PRP injections, and it achieved clinically relevant and statistically significant improvements in patient-reported pain and functional outcomes. These encouraging findings should pave the way toward future controlled, randomized, prospective clinical trials on larger patient populations in order to formally determine whether PRP injections could be an alternative to surgical treatment for stable meniscal injuries.

## Figures and Tables

**Table 1:** **Patient demographics.** Values presented as mean ± standard deviation.

Variable	Value
Patients eligible for the study, No.	38
Gender (female/male), No.	8/30
Age at the time of intervention, mean, y	50.68 ± 9.65
Height, cm	1.75 ± 0.66
Weight, kg	79.44 ± 13.25
Body mass index	25.77 ± 3.37
Affected knee (right/left), No.	25/12
Affected meniscus (medial/lateral), No.	32/6
Meniscus injury grade, No. (%)	
*Grade I*	1 (2.6)
*Grade II*	6 (15.8)
*Grade III*	31 (81.6)

**Table 2:** **Clinicofunctional relevant data of patients undergoing platelet-rich plasma injections.** B, body; PH, posterior horn; A, anterior horn.

Case NO.	Age (YR)	Meniscal Location (Knee, Meniscus; Grade, Tear Location)	Duration of Symptoms, Days	Koos Score Pre-Intervention	Follow-Up, Days	Level of Satisfaction	Koos Score Post-Intervention
**1**	49	Right	30	63	56	Very satisfied	83
		Medial; Grade III, PH					
**2**	47	Left	45	50	56	Satisfied	83
		Medial; Grade III, PH					
**3**	48	Right	150	80	56	Satisfied	89
		Medial; Grade II, PH					
**4**	53	Right	90	81	56	Satisfied	86
		Medial; Grade III, PH					
**5**	52	Left	100	60	56	Very satisfied	81
		Medial; Grade III, PH					
**6**	48	Right	365	80	56	Satisfied	89
		Medial; Grade III, PH					
**7**	48	Right	25	88	56	Very satisfied	91
		Medial; Grade III, PH					
**8**	46	Right	190	35	190	Very satisfied	96
		Medial; Grade III, B + PH					
**9**	40	Right	93	54	93	Very satisfied	89
		Medial; Grade III, B + PH					
**10**	69	Right	78	40	78	Very satisfied	90
		Medial; Grade II, PH					
**11**	51	Left	73	57	73	Very satisfied	100
		Medial; Grade III, B + PH					
**12**	61	Right	57	45	57	Very satisfied	80
		Medial; Grade III, B + PH					
**13**	42	Left	59	63	59	Very satisfied	86
		Medial; Grade II, PH					
**14**	51	Right	89	23	89	Satisfied	88
		Medial; Grade III, B + PH					
**15**	66	Right	71	29	71	Satisfied	62
		Medial, Grade III, PH					
**16**	52	Left	129	76	129	Very satisfied	98
		Medial; Grade III, PH					
**17**	60	Right	142	44	142	Very satisfied	92
		Medial, Grade III, PH					
**18**	38	Right	43	23	43	Satisfied	95
		Medial; Grade III, PH					
**19**	42	Right	69	33	69	Very satisfied	96
		Medial; Grade III, PH					
**20**	55	Left	56	15	56	Satisfied	30
		Medial; Grade III, PH					
**21**	52	Both	104	17	104	Very satisfied	96
		Lateral; Grade III, B + PH					
**22**	67	Left	61	13	61	Satisfied	92
		Lateral, Grade III, PH					
**23**	56	Left	96	17	96	Very satisfied	95
		Medial, Grade III, PH					
**24**	32	Left	39	13	39	Very satisfied	96
		Medial; Grade II, PH					
**25**	47	Right	87	66	87	Very satisfied	79
		Medial; Grade III, PH					
**26**	39	Right	73	63	73	Satisfied	83
		Medial; Grade III, PH					
**27**	49	Right	74	18	74	Satisfied	93
		Medial; Grade III, PH					
**28**	72	Left	96	27	96	Very satisfied	74
		Medial, Grade II, B + PH					
**29**	48	Right	46	23	46	Very satisfied	83
		Both; Grade III, PH (medial), B (lateral)					
**30**	57	Right	89	24	89	Very satisfied	50
		Medial; Grade III, PH					
**31**	57	Right	45	27	45	Very satisfied	93
		Medial, Grade I, PH					
**32**	49	Right	67	22	67	Very satisfied	95
		Lateral; Grade II, PH					
**33**	51	Right	56	63	56	Satisfied	93
		Lateral; Grade III, PH					
**34**	49	Right	134	11	134	Very satisfied	90
		Medial; Grade III, PH					
**35**	40	Right	43	62	43	Satisfied	80
		Lateral; Grade III, AH					
**36**	48	Left	92	24	92	Very satisfied	97
		Medial; Grade III, B + PH					
**37**	29	Left	93	29	93	Very satisfied	87
		Medial; Grade III, PH					
**38**	66	Right	49	34	49	Very satisfied	86
		Medial, Grade III, PH					

**Table 3:** **Comparison of patients’ outcomes pre- and post-intervention.** Numerical Pain Rating Index Scale (NPRS); Knee Injury Osteoarthritis Outcome Score (KOOS); standard deviation (SD); standard Error (SE).

*Outcome*	Pre-Intervention		Post-Intervention		*p*-Value
	*Mean*	*SD/SE*	*Mean*	*SD/SE*	
*NPRS*	5.86	1.91/0.31	1.5789	1.34/1.34	0.000
*Tegner*	3.73	1.67/0.27	4.7368	1.70/0.27	0.001
*KOOS*	41.89	22.96/3.72	85.94	13.50/2.19	0.000
*Feeling thermometer*	67.89	16.42/2.66	86.31	6.74/1.09	0.000

## Data Availability

The datasets used and/or analyzed during the current study are available from the corresponding author on reasonable request.
